# Digital health care solution for proactive heart failure management with the Cordella Heart Failure System: results of the SIRONA first‐in‐human study

**DOI:** 10.1002/ejhf.1870

**Published:** 2020-05-31

**Authors:** Wilfried Mullens, Faisal Sharif, Matthias Dupont, Alexander M.K. Rothman, William Wijns

**Affiliations:** ^1^ Department of Cardiovascular Medicine Ziekenhuis Oost‐Limburg Genk Belgium; ^2^ University Hasselt Hasselt Belgium; ^3^ Department of Cardiology, Galway University Hospital, Saolta Group, CURAM and BioInnovate Ireland National University of Ireland Galway Galway Ireland; ^4^ University of Sheffield and Sheffield Teaching Hospitals NHS Foundation Trust Sheffield UK; ^5^ The Lambe Institute for Translational Medicine and CURAM National University of Ireland Galway Galway Ireland

**Keywords:** Pulmonary artery pressure sensor, Heart failure

## Abstract

**Aims:**

Incorporation of remote monitoring of pulmonary artery pressure and vital signs has been demonstrated to reduce heart failure (HF) hospitalization and all‐cause mortality in selected symptomatic HF patients. The aim of this study is to investigate the safety and accuracy of the new Cordella^TM^ Pulmonary Artery Pressure Sensor (Endotronix, Inc., Chicago, IL, USA) and the usability of the comprehensive Cordella^TM^ Heart Failure System (CHFS).

**Methods and results:**

Multicentre, open‐label, first‐in‐human, feasibility study to evaluate the CHFS and the safety and accuracy of the Cordella™ Pulmonary Artery Pressure Sensor in 15 patients with New York Heart Association class III HF. All patients were successfully implanted with the Cordella Pulmonary Artery Pressure Sensor, without sensor failure. No device system‐related complications, defined as invasive treatment, device explant or death, occurred. The primary efficacy endpoint of a mean pulmonary artery pressure at 90 days was met in all but one patients with a cohort difference of 2.7 mmHg (Cordella Sensor 22.5 ± 11.8 mmHg, Swan–Ganz catheter 25.2 ± 8.5 mmHg). One patient did not go through the 90‐day right heart catheterization for safety reasons. Patient adherence to daily measurement, transmission of vital signs and pulmonary artery pressure sensor readings were recorded 99% of the time.

**Conclusion:**

The initial experience of the CHFS incorporating comprehensive vital signs and pulmonary artery pressure monitoring enables safe and accurate monitoring of HF status.

## Introduction

Incorporation of remotely monitored pulmonary artery pressure (PAP)‐guided treatment algorithm with the aim of maintaining normal PAP range proved to be more effective in reducing heart failure (HF) hospitalizations in selected patients with HF than management based on symptoms and clinical signs.^1^, ^2^ Additionally, it has also been demonstrated that structured and protocol‐driven remote monitoring of daily weights, blood pressure, heart rate and rhythm, oxygen saturations and a self‐rated health status score through a telemedical centre management leads to a reduction in unplanned cardiovascular admissions and all‐cause mortality.^3^ Together, the approved Cordella^TM^ Heart Failure System (CHFS) and the investigational Cordella^TM^ Pulmonary Artery Pressure Sensor (Endotronix, Inc., Chicago, IL, USA) provide comprehensive clinical information including PAP, body weight, blood pressure, heart rate and oxygen saturations of HF patients to their physicians, allowing HF patients to be an integral part of the team and provide a proactive HF management platform. SIRONA sought to examine for the first time the safety and accuracy of the Cordella Pulmonary Artery Pressure Sensor and the usability of the CHFS.

## Methods

### Study design and inclusion criteria

SIRONA (ClinicalTrials.gov Identifier: NCT03375710, CEC Ref. number: 17/061U, date of favourable ethical opinion: 8 November 2017) was a multicentre, open‐label, early feasibility study to evaluate the CHFS and the safety and accuracy of the Cordella™ Pulmonary Artery Pressure Sensor in 15 patients with New York Heart Association (NYHA) class III HF. The study was undertaken in accordance with the Declaration of Helsinki and approved by local ethics committees or institutional review boards. All participants provided written informed consent. Briefly, eligible patients were men or women over 18 years with a diagnosis of NYHA class III HF with reduced or preserved ejection fraction treated for a minimum of 3 months and stable for 1 month prior to enrolment with at least one HF‐related hospitalization or equivalent within the last year. An estimated glomerular filtration rate of ≥30 mL/min/1.73 m^2^ and appropriate pulmonary artery anatomy, as demonstrated by computed tomography pulmonary angiogram (CTPA), were also required.

### Study device

**Figure 1:**
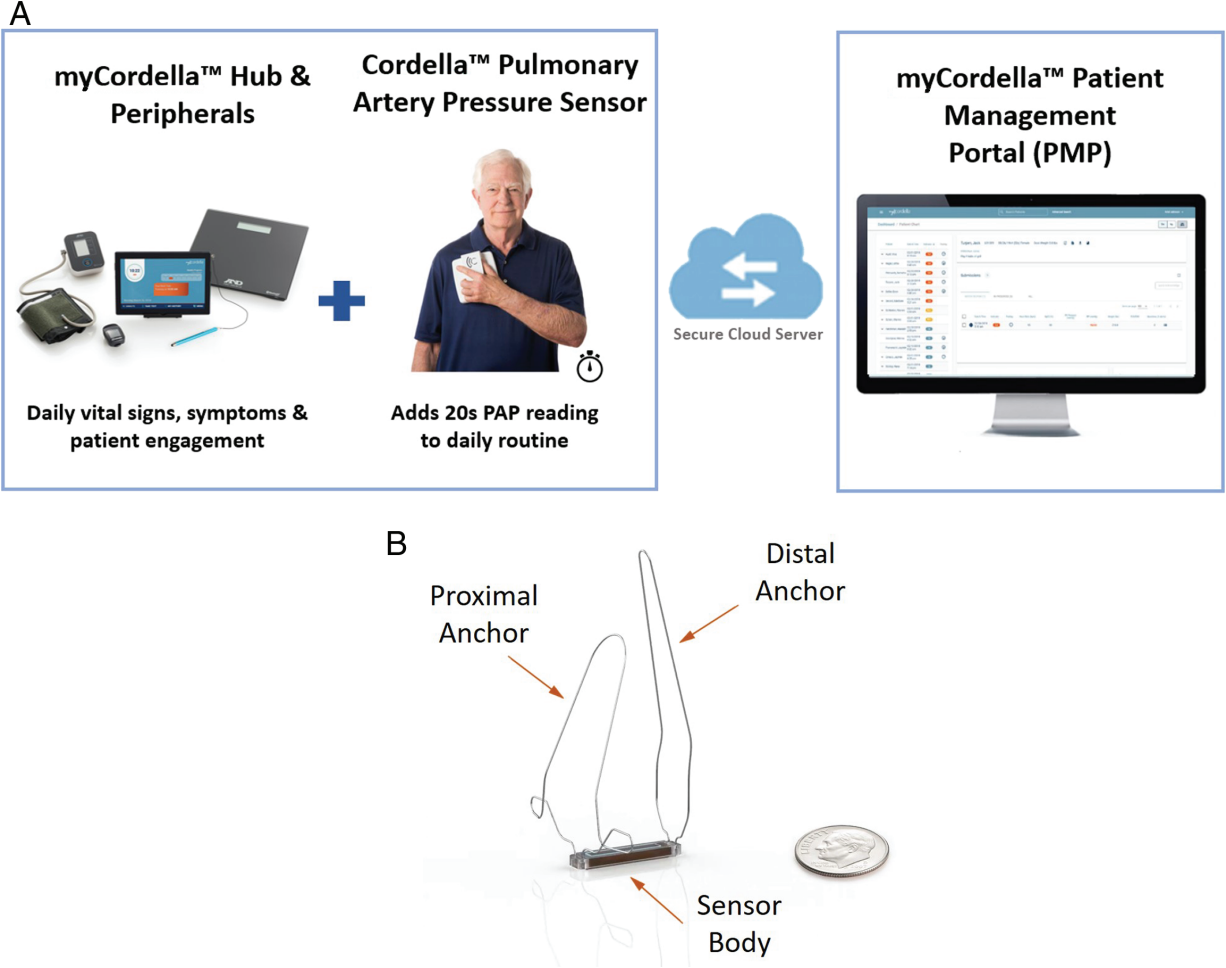
The Cordella™ Heart Failure System. (*A*) The Cordella™ System securely transmits daily health information (weight, blood pressure, saturations, heart rate) as well as pulmonary artery pressure (PAP) data to clinicians from the patient's home. (*B*) The Cordella™ Pulmonary Artery Pressure Sensor.

The CHFS (*Figure* *1*) is a comprehensive digital HF management technology that measures, records, and transmits vital signs (blood pressure, heart rate, weight, oxygen saturations) and PAP data from the patients' home to the clinical teams for proactive management of patients with HF. Data are transmitted for review by the clinical team on the web‐based Patient Management Portal. Quality of life was measured with the Kansas City Cardiomyopathy Questionnaire (KCCQ) answered by the patients directly on a patient application, that is part of the CHFS.

**Figure 2:**
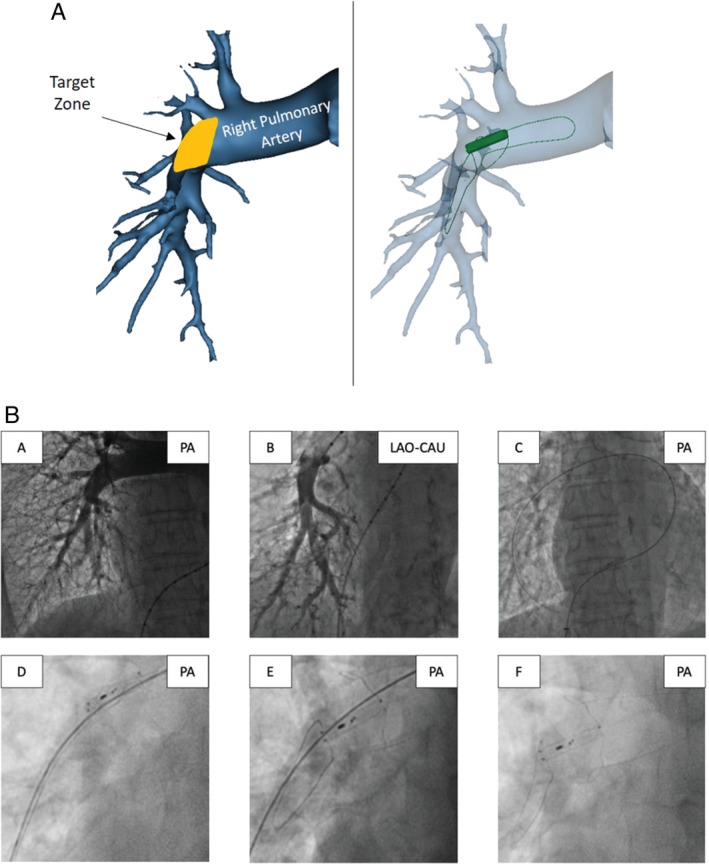
The Cordella^TM^ Pulmonary Artery Pressure Sensor. (*A*) Schematic of the sensor implanted in the right pulmonary artery in anterior‐posterior view: target implant zone and three‐dimensional reconstruction of the Cordella Sensor deployed at the target zone. (*B*) Implantation technique. Pulmonary angiography in the (A) antero‐posterior (PA) and (B) left anterior oblique caudal (LAO‐CAU) views. (C) 0.025” × 260 cm Amplatz extra support wire in a side branch of the right pulmonary artery over which the delivery system is advanced, (D) positioning of the Cordella Sensor in the right pulmonary artery, (E) withdrawal of release wires allows nitinol anchors to expand securing the sensor within the vessel, and (F) final positioning of the Cordella Sensor in the right pulmonary artery.

The Cordella Sensor is implanted in the right pulmonary artery (*Figure* *2A*), and PAP is measured daily by a wireless handheld reader (myCordella™ Patient Reader). Patients are prompted to take daily readings using their tablets, which is part of the system, based on the physician‐scheduled time. However, patients can take as many ad‐hoc readings as desired any time, which will also be displayed in the system. The system takes an 18 s reading of the PAP and the mean PAP is calculated across the full 18 s with free breathing. Briefly, the patient undocks the reader from its docking station, holds it against their chest with one hand for 18 s while seated, and re‐docks in response to an audio cue. Data (mean and beat‐to‐beat) are then transmitted for review by the clinical team on the web‐based Patient Management Portal. A change of nitinol anchors was developed during the study to accommodate a broader range of patient and vessel anatomy and sizes and to improve stability of the device. The iteration lengthened and angulated the distal anchor to stabilize the device on the inferior‐posterior inflection of the pulmonary artery. Seven patients were implanted with the initial anchor design and eight patients with the updated anchor design.

### Placement of the Cordella™ Pulmonary Artery Pressure Sensor

After a minimum of 5 days of compliant use of the CHFS, patients underwent right heart catheterization and implantation of the Cordella™ Sensor (*Figure* *2B*). In SIRONA, only patients with a favourable anatomy based upon computed tomography scan were included due to the size requirements of the initial anchor design. A 14 Fr introducer was inserted into the right femoral vein and right heart catheterization undertaken using a 7.5 Fr Swan–Ganz catheter (Edwards Lifesciences). Through the distal lumen of the Swan–Ganz catheter a 0.025″ x 260 Amplatz extra support wire (Cook) was positioned in the right pulmonary artery and the Swan–Ganz catheter exchanged for a 5 Fr Straight Flush angiographic catheter (Merit). Pulmonary angiography was performed in the antero‐posterior and left anterior oblique caudal views (40 mL, 2 s, 600 psi). The 0.025″ × 260 Amplatz extra support wire was then positioned in a right lower lobe branch of the pulmonary artery (A8–10), and the Straight Flush catheter exchanged for the delivery system. The Cordella Sensor, pre‐mounted on the delivery system, was advanced through the right heart and positioned at the inferior‐posterior inflection of the right pulmonary artery. Position was confirmed by hand injection of contrast through the side‐arm of the delivery system. Withdrawal of the release wires served to free the self‐aligning nitinol anchors and secure the Cordella Sensor in the pulmonary artery. Following implantation, the system was calibrated to a reference fluid‐filled pressure measurement using the CalEQ (calibration system).

Patients remained in hospital one night post‐procedure for observation. Following implantation, daily PAP readings were performed while seated, holding the wireless handheld sensor reading device (myCordella™ Patient Reader) against the right pectoral region for 20 s and vital signs (blood pressure, heart rate, weight, oxygen saturations) were measured using the CHFS. Data were transmitted to the myCordella™ Hub, which guides patients on use of the peripherals, asks the patient health‐related questions, and facilitates patient–clinician communication by re‐transmitting data to the myCordella™ Patient Management Portal (*Figure* *1*).

### Follow‐up and endpoints

Post‐procedure follow‐up was scheduled at 1, 3, 6, 9, 12, 18, and 24 months. History and clinical examination were recorded and laboratory assessments undertaken at each visit. Right heart catheterization was performed at baseline and at 3‐ and 12‐month follow‐up in the supine position. The clinicians were blinded to PAP the first 90 days until the accuracy check at 90‐day right heart catheterization.

The primary safety endpoint was freedom from device‐related adverse events through 30 days post‐procedure and pressure sensor failure. The primary efficacy endpoint was accuracy of the Cordella Sensor PAP measurements, compared to fluid‐filled catheter 90 days post‐implant. Published data estimate the error of the fluid‐filled catheter as ± 4.0 mmHg. For the Cordella system to be at least as accurate as fluid‐filled, the combined system error has to be less than ± 5.6 mmHg (root square sum of fluid‐filled and Cordella error) with a 95% confidence interval for the total system of ± 11 mmHg. Thus, the pre‐specified primary endpoint was fluid‐filled catheter ± 11 mmHg, with a difference of ± 11 mmHg; in order to meet this endpoint the Cordella Sensor has to be as accurate as a standard‐of‐care fluid‐filled catheter which is ± 4 mmHg.

Secondary efficacy endpoints included change in PAP pre‐ and post‐implant, frequency of HF hospitalization or equivalent, change in quality of life (KCCQ and EQ‐5D‐5L) and adherence to myCordella™ Heart Failure System measurements. An independent data safety and monitoring board reviewed all potential procedure‐related serious adverse events and adverse events.

### Statistical analysis

The Cordella™ system accuracy was compared to the accuracy of standard‐of‐care commercial products that use fluid‐filled invasive catheters to measure PAP, which have an accuracy of ± 4 mmHg. If ± 4 mmHg is assumed for both Cordella™ and fluid‐filled invasive catheters, then the total system accuracy must be less than ± 5.6 mmHg. This total system accuracy (± 5.6 mmHg) translates into 95% of all Cordella readings must be within ± 11 mmHg of fluid‐filled catheter.^4^

$$\sigma_{\text{system}}=\sqrt{{\sigma_{\text{fluid}-\text{filled}}}^{2}+{\sigma_{\mathrm{PA}\;\text{Sensor}}}^{2}}$$

$$5.6\;\text{mmHg}=\sqrt{4^{2}+4^{2}}$$

Cordella™ readings were compared to reference readings using the Bland–Altman method. A Bland–Altman plot displayed the difference between the measurements from a standard right heart catheterization and the Cordella™ device (y‐axis), vs. the average of these two measurements (x‐axis) for a measurement taken within 24 h of implant and 90 days. This variance corresponds to the documented accuracy of typical standard‐of‐care fluid‐filled invasive catheter pressure measurement systems. Statistical analyses were performed in accordance with the pre‐specified statistical analysis plan, and SAS version 9.3 (SAS Institute Inc., Cary, NC, USA) or higher was used to analyse the data.

## Results

**Figure 3:**
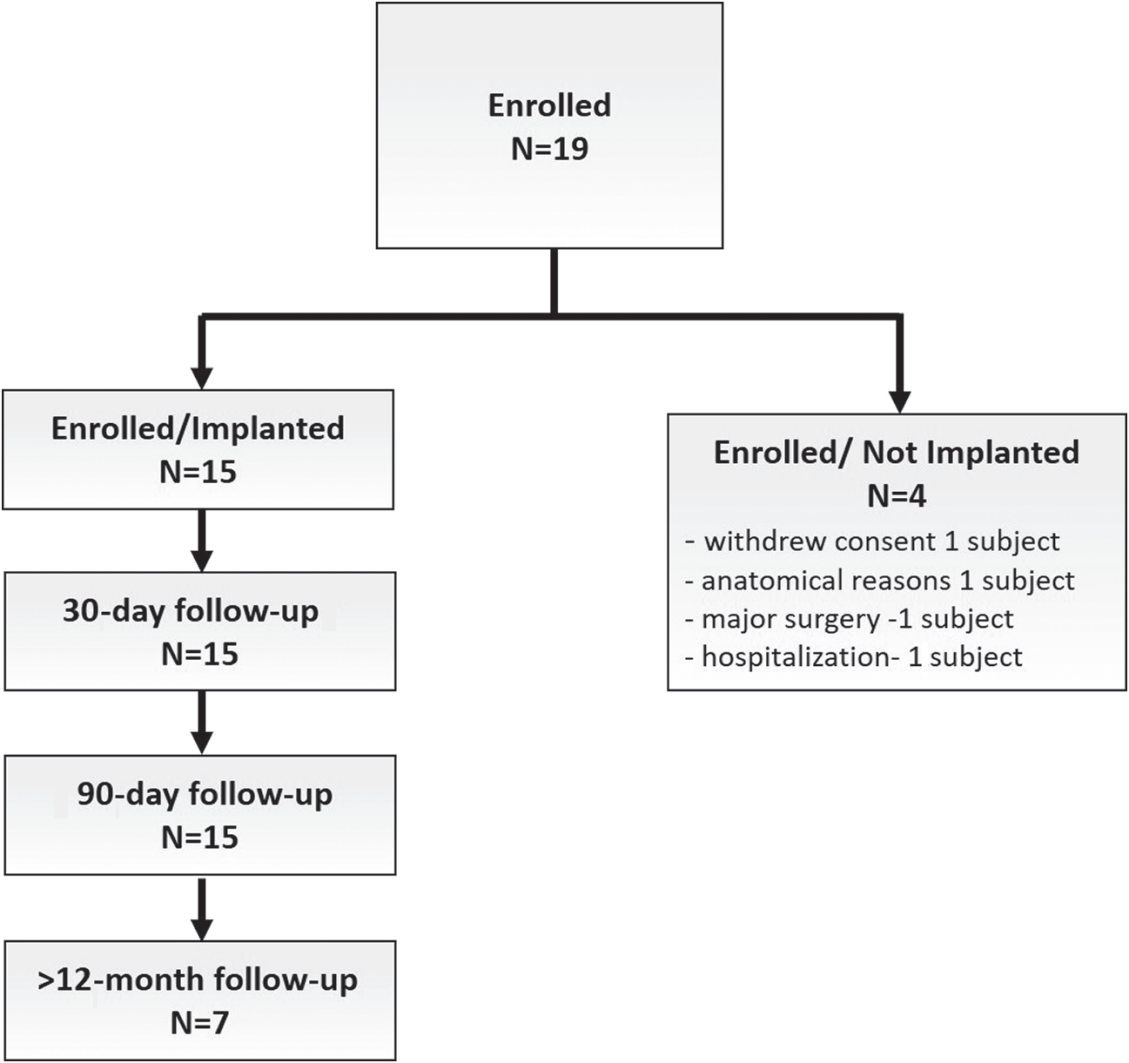
Enrolment diagram (consort diagram).

Between December 2017 and February 2019, 19 patients with HF stable on therapy with HF‐related hospitalization within the preceding 12 months were enrolled in the SIRONA study. After a minimum 5‐day period adherent to daily vital sign collection using the CHFS and a qualifying CTPA and pulmonary angiogram, 15 patients were scheduled to undergo implantation of the Cordella Pulmonary Artery Pressure Sensor (*Figure* *3*). All patients were in NYHA class III; the mean age was 71.4 years (range 47.6–86.5), mean body mass index was 29 (17–39) kg/m^2^, 10 (67%) were male and 4 (53%) had an ejection fraction >40% (*Table* *1*).

**Table 1:** Patient demographics

Implanted population, *n*	15
Demographics	
Age, years, mean (range)	71.44 (47.6–86.5)
Male sex, % (*n*)	67 (10)
Caucasian race, % (*n*)	100 (15)
Height, m, mean (range)	1.673 (1.48–1.86)
Weight, kg, mean (range)	78.55 (44.8–115.1)
BMI, kg/m^2^, mean (range)	29 (17–39)
Ejection fraction >40%, % (*n*)	53 (8)
MI history, % (*n*)	47 (7)
NYHA class III, % (*n*)	100 (15)
Comorbidities, % (*n*)	
Diabetes mellitus	27 (4)
Hypertension	67 (10)
COPD	20 (3)
Atrial flutter/fibrillation	60 (9)
CAD	47 (7)

BMI, body mass index; CAD, coronary artery disease; COPD, chronic obstructive pulmonary disease; MI, myocardial infarction; NYHA, New York Heart Association.

All 15 patients were successfully implanted with the Cordella Pulmonary Artery Pressure Sensor. Four adverse events, related to the use of the CHFS, were reported: one sensor was dislodged from the target location of deployment into the main pulmonary artery during withdrawal of the delivery system (not requiring any treatment and no compromise to sensor performance); transient complete heart block was identified in a single patient as the sensor passed through the right heart; post‐procedure minor haemoptysis was reported in two patients (*Table* *2*). All events resolved without clinical sequelae or impairment of device function. No device system‐related complication, defined as invasive treatment, device explant or death, occurred. No sensor failure occurred within 90 days. The only serious adverse event within 90 days was one HF hospitalization.

**Table 2:** Adverse events related to the use of the Cordella Heart Failure System

Adverse event	No. events	Therapy	Outcome
Total adverse events	4		
Dislodgement of sensor	1	None	Recovered without sequelae with no impact on pulmonary artery pressure readings
Procedural complete heart block	1	None	Recovered without sequelae
Haemoptysis	2	None (*n* = 1) Single dose of protamine (*n* = 1)	Recovered without sequelae

**Figure 4:**
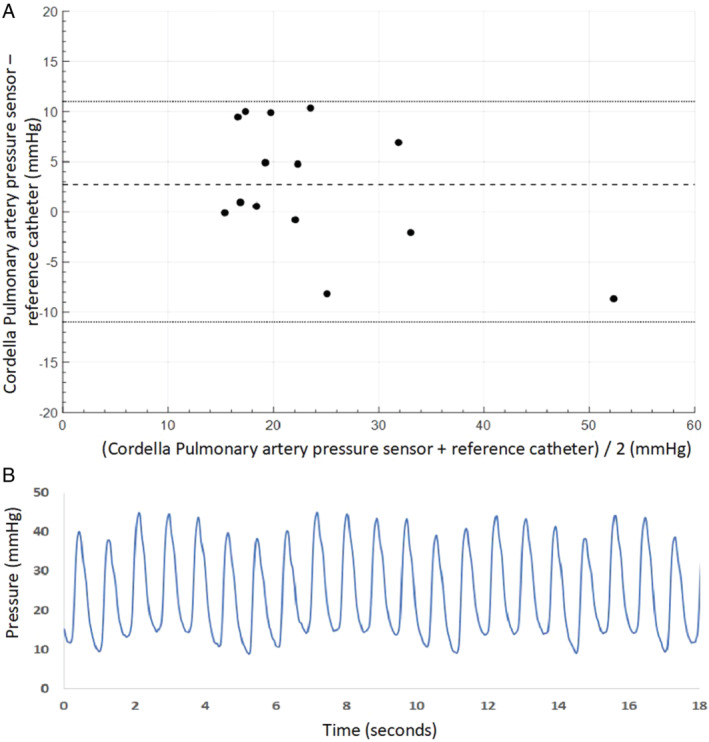
Pulmonary artery pressure measurement: (*A*) Bland–Altman plot of mean pulmonary artery pressure 90 days following implantation. The Cordella Pulmonary Artery Pressure Sensor readings are compared to fluid‐filled catheter. (*B*) The Cordella Pulmonary Artery Pressure Sensor‐derived waveform.

**Figure 5:**
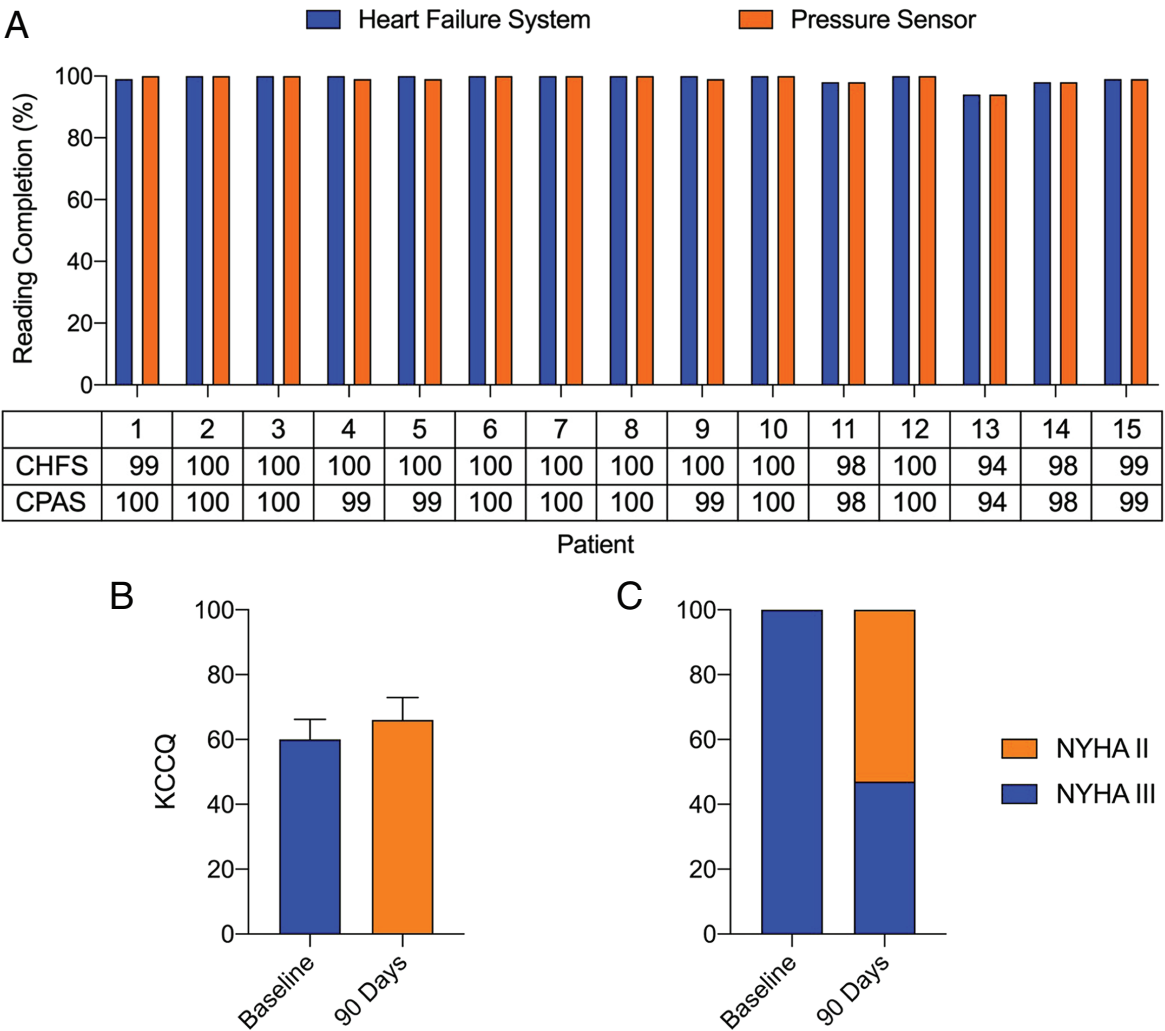
Patient adherence, quality of life and New York Heart Association (NYHA) classification. (*A*) Patient adherence with daily Cordella^TM^ Heart Failure System (CHFS) and Cordella™ Pulmonary Artery Pressure Sensor (CPAS) readings over the first 90 days following implantation. (*B*) Kansas City Cardiomyopathy Questionnaire (KCCQ) score at baseline and 90 days following implantation. (*C*) NYHA classification at baseline and 90 days following implantation.

At 90 days post‐implantation, PAP values measured by the Cordella Sensor and right heart catheterization were well matched. The primary efficacy endpoint of a mean PAP was met in all patients with a cohort difference of 2.7 mmHg (Cordella Sensor 22.5 ± 11.8 mmHg, Swan–Ganz catheter 25.2 ± 8.5 mmHg). Systolic and diastolic PAP values were also well matched over a clinically relevant pressure range (online supplementary *Table* *S1* and *Figure* *4*). The one patient who had a device dislodgement during the implant procedure did not go through the 90‐day right heart catheterization procedure as we did not want to take the risk to further dislodge the sensor. Over the first 90 days following implantation, patient adherence to daily measurement and transmission of vital signs was 99% and PAP sensor readings was 99% corroborated with improvements in KCCQ score and NYHA classification (*Figure* *5*).

## Discussion

SIRONA was a first‐in‐human multicentre clinical study combining the CHFS and Cordella Sensor to provide PAP, weight, blood pressure, heart rate and oxygen saturations to patients and physicians through a remote monitoring system in ambulatory symptomatic HF patients. The study demonstrated that implantation of the Cordella Sensor was feasible and safe with excellent accuracy of the Cordella Sensor PAP measurements, compared to fluid‐filled catheter at 3‐month follow‐up. Additionally, the user‐friendly patient interface with measurement of PAP through a handheld device facilitated patient engagement and empowerment, resulting in high compliance during the study period. As such, the CHFS incorporating vital signs as well as PAP monitoring is positioned to enable comprehensive proactive HF monitoring and management.

Heart failure hospitalization rates remain unacceptably high and are associated with substantial patient, caregiver, and economic costs. Randomized controlled trials of non‐invasive telemedical systems compared to routine follow‐up, have mostly failed to demonstrate reduced rates of hospitalization, which probably relates to the limitations of the signals measured as well as the inadequate reaction to the signals. As such, a recent clinical practice update on HF by the Heart Failure Association of the European Society of Cardiology only recommended that home telemonitoring ‘may be used’ to enhance patient education and motivation and delivery of care but must be adapted to work in synergy with existing healthcare provision, providing the cost/benefit needs are adequately assessed.^5^ In contrast, remote PAP‐guided management has demonstrated to reduce HF hospitalization and all‐cause mortality in selected symptomatic HF patients in the CHAMPION (CardioMEMS Heart Sensor Allows Monitoring of Pressure to Improve Outcomes in NYHA Class III Heart Failure Patients) trial, regardless of ejection fraction.^1^, ^6^ However, the cost and technical limitations of the current system hamper widespread implementation of this technology.

The combination of both invasive and non‐invasive monitoring systems with an easy‐to‐use patient–health care provider interface has never been tested. Here we report the feasibility, safety and compliance of this combined comprehensive telemonitoring system together with a PAP sensor. Importantly, data were easy to measure by the patient and sent through a user‐friendly patient experience, enabling patient engagement and empowerment. This also allows a bidirectional line of communication between patient and heart care providers. As illustrated in the patient profile (online supplementary *Figure* *S1*), the integration of clinical signs and PAP allows better phenotyping of HF, leading to a more targeted HF approach where other approaches only using one of both systems might fail.

The Cordella Sensor requires no leads or batteries and is concurrently powered and interrogated via an external antenna in the handheld reader. It is easily implanted into a branch of the right pulmonary artery during right heart catheterization, using a specialized delivery system with the possibility of periprocedural injection of dye, making selective cannulation of the desired branch possible, under local anaesthesia. The design of the anchors allows the sensor to be placed in an anterior branch of the right pulmonic artery, which together with its microelectronic mechanical system designed for narrowband radiofrequency energizing and sensing, makes an anterior reading of the device possible, thus allowing PAP measurements in a seated position. Electromagnetic coupling is achieved by an external antenna, which is incorporated in the handheld device, which is held against the anterior chest wall, further improving patient compliance. Pressure applied to the sensor causes deflections of the pressure‐sensitive surface, resulting in a characteristic shift in the resonant frequency.

Based upon the promising results of SIRONA, a robust clinical programme is currently underway to demonstrate the safety and efficacy of the Cordella Pulmonary Artery Pressure Sensor in SIRONA 2 (NCT03375710) as well as PROACTIVE‐HF (NCT04089059).

## Conclusion

The CHFS incorporating comprehensive vital signs as well as PAP monitoring enables safe and accurate monitoring of HF status.

### Funding

The study was funded by Endotronix Inc. Independent data collection, monitoring and data analysis were undertaken by independent contract research organizations (CROs). AR is supported by a Wellcome Trust Clinical Research Career Development Fellowship (206632/Z/17/Z) [Correction added on 18 August 2020, after first online publication: Funding section has been updated to cite ‘Wellcome Trust’ as funder of the author Alexander Rothman.]. All authors had access to data and were responsible for the decision to submit. None of the Authors received any personal financial compensation of Endotronix Inc.

**Conflict of interest:** A.M.K.R. reports research support from Abbott, Medtronic, Actelion, Novartis, SoniVie, Endotronix. All other authors have nothing to disclose.

## Supporting information


**Figure S1.** Single patient‐derived data following informed consent, study enrolment and pulmonary artery pressure sensor implantation. (1) Improved therapeutic adherence to guideline‐directed medical therapy reduced pulmonary artery pressure. (2) Transition from angiotensin‐converting enzyme inhibitor to sacubitril/valsartan reduced pulmonary artery pressure. (3) Increase in pulmonary artery pressure and decreased weight preceded hospital admission with a respiratory infection.

**Table S1.** Pulmonary artery pressure during 90‐day right heart catheterization.

## References

[1] Abraham WT , Adamson PB , Bourge RC , Aaron MF , Costanzo MR , Stevenson LW , Strickland W , Neelagaru S , Raval N , Krueger S , Weiner S , Shavelle D , Jeffries B , Yadav JS ; CHAMPION Trial Study Group . Wireless pulmonary artery haemodynamic monitoring in chronic heart failure: a randomised controlled trial. Lancet 2011;377:658–666. 21315441 10.1016/S0140-6736(11)60101-3

[2] Constanzo M , Stevenson LW , Adamson PB , Deasi AS , Heywood JT , Bourge RC , Bauman J , Abraham WT . Interventions linked to decreased heart failure hospitalizations during ambulatory pulmonary artery pressure monitoring. JACC Heart Fail 2016;4:333–344. 26874388 10.1016/j.jchf.2015.11.011

[3] Koehler F , Koehler K , Deckwart O , Prescher S , Wegscheider K , Kirwan BA , Winkler S , Vettorazzi E , Bruch L , Oeff M , Zugck C , Doerr G , Naegele H , Störk S , Butter C , Sechtem U , Angermann C , Gola G , Prondzinsky R , Edelmann F , Spethmann S , Schellong SM , Schulze PC , Bauersachs J , Wellge B , Schoebel C , Tajsic M , Dreger H , Anker SD , Stangl K . Efficacy of telemedical interventional management in patients with heart failure (TIM‐HF2): a randomised, controlled, parallel‐group, unmasked trial. Lancet 2018;392:1047–1057. 30153985 10.1016/S0140-6736(18)31880-4

[4] Abraham WT , Adamson PB , Hasan A , Bourge RC , Pamboukian SV , Aaron MF , Raval NY . Safety and accuracy of a wireless pulmonary artery pressure monitoring system in patients with heart failure. Am Heart J 2011;161:558–566. 21392612 10.1016/j.ahj.2010.10.041

[5] Seferovic P , Ponikowski P , Anker SD , Bauersachs J , Chioncel O , Cleland JG , Boer RA , Drexel H , Ben Gal T , Hill L , Jaarsma T , Jankowska EA , Anker MS , Lainscak M , Lewis BS , McDonagh T , Metra M , Milicic D , Mullens W , Piepoli MF , Rosano G , Ruschitzka F , Volterrani M , Voors AA , Filippatos G , Coats AJ . Clinical practice update on heart failure 2019: pharmacotherapy, procedures, devices and patient management. An expert consensus meeting report of the Heart Failure Association of the European Society of Cardiology. Eur J Heart Fail 2019;21:1169–1186. 31129923 10.1002/ejhf.1531

[6] Heywood JT , Jermyn R , Shavelle D , Abraham WT , Bhimaraj A , Bhatt K , Sheikh F , Eichorn E , Lamba S , Bharmi R , Agarwal R , Kumar C , Stevenson LW . Impact of practice‐based management of pulmonary artery pressures in 2000 patients implanted with the CardioMEMS sensor. Circulation 2017;135:1509–1517. 28219895 10.1161/CIRCULATIONAHA.116.026184

