# Supplementary figures and images for: Digital health care solution for proactive heart failure management with the Cordella Heart Failure System: results of the SIRONA first‐in‐human study
Source: Eur J Heart Fail. 2020 May 31;22(10):1912–9. doi: 10.1002/ejhf.1870 (PMC7687200; doi:10.1002/ejhf.1870)

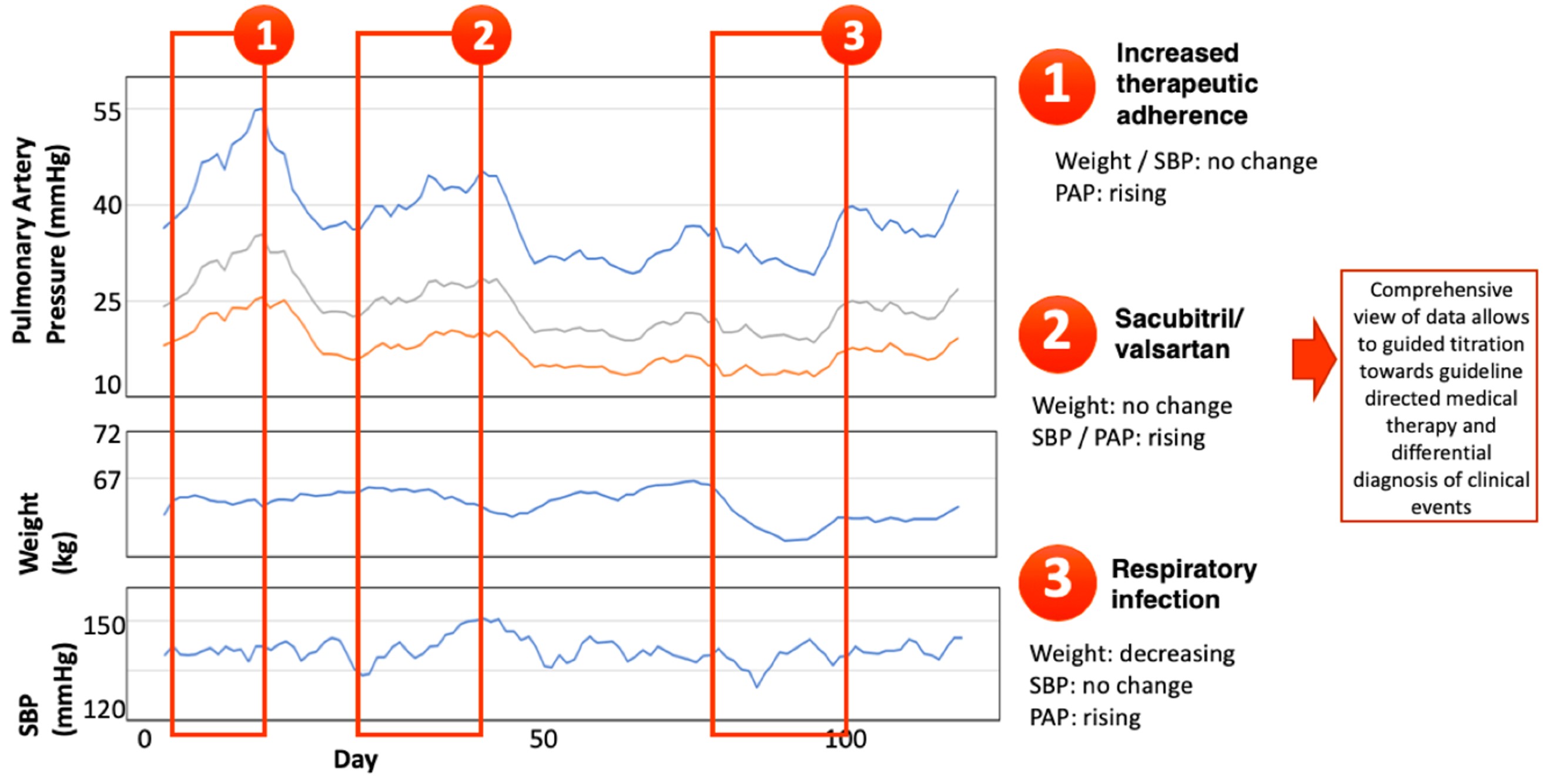

Supplement: Supplementary file 1 — Figure S1. Single patient‐derived data following informed consent, study enrolment and pulmonary artery pressure sensor implantation. (1) Improved therapeutic adherence to guideline‐directed medical therapy reduced pulmonary artery pressure. (2) Transition from angiotensin‐converting enzyme inhibitor to sacubitril/valsartan reduced pulmonary artery pressure. (3) Increase in pulmonary artery pressure and decreased weight preceded hospital admission with a respiratory infection. [file EJHF-22-1912-s001.jpg]
