# Supplementary material for: Digital health care solution for proactive heart failure management with the Cordella Heart Failure System: results of the SIRONA first‐in‐human study
Source: Eur J Heart Fail. 2020 May 31;22(10):1912–9. doi: 10.1002/ejhf.1870 (PMC7687200; doi:10.1002/ejhf.1870)
Supplement: Supplementary file 2 — Table S1. Pulmonary artery pressure during 90‐day right heart catheterization. [file EJHF-22-1912-s002.docx]

Table 1. Pulmonary artery pressure during 90 day right heart catherization.

|  | sPAP | | mPAP | | dPAP | |
| --- | --- | --- | --- | --- | --- | --- |
|  | Cordella™ Sensor | Swan-Ganz | Cordella ™ Sensor | Swan-Ganz | Cordella™ Sensor | Swan-Ganz |
| n | 14 | 14 | 14 | 14 | 14 | 14 |
| Mean (SD) | 38.0 (14.4) | 43.9 (13.5) | 22.5 (11.8) | 25.2 (8.5) | 14.7 (10.8) | 15.9 (7.1) |
| Median | 33.9 | 42 | 18.2 | 22 | 10.3 | 15 |
| Min, Max | 25.2, 77.5 | 26.0, 72.0 | 11.9, 56.6 | 15.3, 48 | 4.4, 46.2 | 7.0, 36.0 |
| Mean Difference |  | | 2.7 | |  |  |
| 95% Confidence Interval Limit |  | | -11.1, 16.5 | |  |  |
